# Spectral Diffusion Analysis of Intravoxel Incoherent Motion MRI in Cerebral Small Vessel Disease

**DOI:** 10.1002/jmri.26920

**Published:** 2019-09-04

**Authors:** Sau May Wong, Walter H. Backes, Gerhard S. Drenthen, C. Eleana Zhang, Paulien H.M. Voorter, Julie Staals, Robert J. van Oostenbrugge, Jacobus F.A. Jansen

**Affiliations:** ^1^ Department of Radiology & Nuclear Medicine Maastricht University Medical Centre Maastricht the Netherlands; ^2^ Department of Neurology Maastricht University Medical Centre Maastricht the Netherlands; ^3^ Department of School for Mental Health and Neuroscience (MHeNs) Maastricht University Medical Centre Maastricht the Netherlands; ^4^ Cardiovascular Research Institute Maastricht (CARIM) Maastricht University Medical Centre Maastricht the Netherlands; ^5^ Department of Electrical Engineering Eindhoven University of Technology Eindhoven the Netherlands; ^6^ Department of Biomedical Engineering Eindhoven University of Technology Eindhoven the Netherlands

**Keywords:** cerebral small vessel disease, MRI, glymphatic system, diffusion magnetic resonance imaging

## Abstract

**Background:**

Cerebral intravoxel incoherent motion (IVIM) imaging assumes two components. However, more compartments are likely present in pathologic tissue. We hypothesized that spectral analysis using a nonnegative least‐squares (NNLS) approach can detect an additional, intermediate diffusion component, distinct from the parenchymal and microvascular components, in lesion‐prone regions.

**Purpose:**

To investigate the presence of this intermediate diffusion component and its relation with cerebral small vessel disease (cSVD)‐related lesions.

**Study Type:**

Prospective cross‐sectional study.

**Population:**

Patients with cSVD (*n* = 69, median age 69.8) and controls (*n* = 39, median age 68.9).

**Field Strength/Sequence:**

Whole‐brain inversion recovery IVIM acquisition at 3.0T.

**Assessment:**

Enlarged perivascular spaces (PVS) were rated by three raters. White matter hyperintensities (WMH) were identified on a fluid attenuated inversion recovery (FLAIR) image using a semiautomated algorithm.

**Statistical Tests:**

Relations between IVIM measures and cSVD‐related lesions were studied using the Spearman's rank order correlation.

**Results:**

NNLS yielded diffusion spectra from which the intermediate volume fraction *f*_int_ was apparent between parenchymal diffusion and microvasular pseudodiffusion. WMH volume and the extent of MRI‐visible enlarged PVS in the basal ganglia (BG) and centrum semiovale (CSO) were correlated with *f*_int_ in the WMHs, BG, and CSO, respectively. *f*_int_ was 4.2 ± 1.7%, 7.0 ± 4.1% and 13.6 ± 7.7% in BG and 3.9 ± 1.3%, 4.4 ± 1.4% and 4.5 ± 1.2% in CSO for the groups with low, moderate, and high number of enlarged PVS, respectively, and increased with the extent of enlarged PVS (BG: *r* = 0.49, *P* < 0.01; CSO: *r* = 0.23, *P* = 0.02). *f*_int_ in the WMHs was 27.1 ± 13.1%, and increased with the WMH volume (*r* = 0.57, *P* < 0.01).

**Data Conclusion:**

We revealed the presence of an intermediate diffusion component in lesion‐prone regions of cSVD and demonstrated its relation with enlarged PVS and WMHs. In tissue with these lesions, tissue degeneration or perivascular edema can lead to more freely diffusing interstitial fluid contributing to *f*_int_.

**Level of Evidence:** 2

**Technical Efficacy:** Stage 2

J. Magn. Reson. Imaging 2020;51:1170–1180.

NONINVASIVE IMAGING can play an important role in the management of cerebral small vessel disease (cSVD), as it can provide in vivo pathophysiological information, without having to rely on histopathology. In recent years, intravoxel incoherent motion (IVIM) magnetic resonance imaging (MRI) has received growing attention in brain research.^1^ This noninvasive diffusion‐weighted imaging technique is widely available on clinical scanners and is suggested to measure both diffusion and perfusion properties of cerebral tissue.

Traditionally, two diffusion components are considered to be present in brain IVIM, ie, parenchymal and microvascular components, which arise from water diffusion in the parenchyma and flow‐mediated intravascular (pseudo) diffusivity of microvascular blood,^2^ respectively. However, in cSVD more components with distinct diffusion properties can be hypothesized to be present in disordered tissues. For instance, aberrant amounts of interstitial fluid (ISF), which can diffuse more freely, can be observed in lesion‐prone tissue regions in cSVD (ie, where white matter hyperintensities [WMHs] and enlarged perivascular spaces [PVS] may occur) (Fig. 1).^3^ Recently, the nonnegative least‐squares (NNLS) method, which has no constraints on the number of components, has been proposed for the analysis of the IVIM signal, yielding a diffusion spectrum.^4^, ^5^ Values for both the parenchymal diffusivity and microvascular perfusion can be detected in this spectrum.

**Figure 1:**
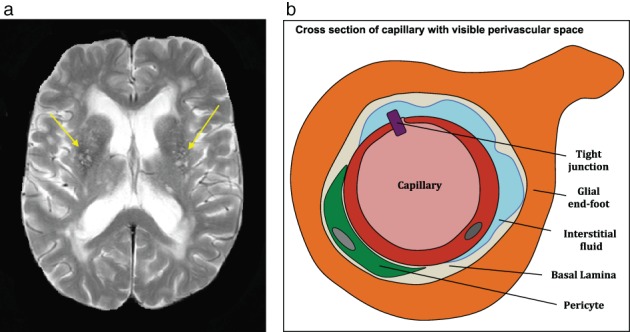
A T_2_‐weighted image **(a)** showing enlarged perivascular spaces (yellow arrows) and a schematic representation of a cross‐section of a capillary with enlarged perivascular space **(b)**. Perivascular spaces are conduits surrounding perforating vessels containing interstitial fluid and its visible enlargements are often observed in the basal ganglia and centrum semiovale.

In lesion‐prone regions, we hypothesized that an additional component, distinctive from both the parenchymal diffusion and microvascular perfusion components, can be visualized in the diffusion spectrum. The present study was set up to improve IVIM analysis in pathologic cerebral tissue by employing spectral analysis using NNLS to investigate the presence of an additional component in the diffusion spectrum and its potential relation with cSVD‐related lesions, in terms of WMHs and MRI‐visible enlarged PVS.

## Materials and Methods

### *Standard Protocol Approval*

This is a prospective study and was registered at trial register NTR3786 and approved by the local Medical Ethics Committee. The study was conducted in accordance with the Declaration of Helsinki. All participants gave written informed consent prior to inclusion.

### *Study Population*

We studied 108 participants, including patients with clinically overt cSVD (first ever lacunar stroke or mild vascular cognitive impairment (mVCI))^6^ (*n* = 69, 69.8 ± 10.6 years [mean ± SD], 59.4% male) and age‐ and sex‐matched healthy controls (*n* = 39, 68.9 ± 11.6 years, 59.0% male). Lacunar stroke patients were recruited from the stroke unit. Lacunar stroke was defined as an acute stroke syndrome with a compatible recent small subcortical infarct on brain MRI.^6^, ^7^ If no such lesion was visible on imaging, established criteria for lacunar stroke syndrome were used. Exclusion criteria included a potential cardiac embolic source (eg, atrial fibrillation) or symptomatic carotid stenosis of ≥50%. Stroke patients were included at least 3 months poststroke to avoid acute stroke changes. Patients with mVCI were recruited from the outpatient clinic of the Department of Neurology and from the Memory Clinic. Criteria of mVCI were met when patients had 1) subjective complaints of cognitive functioning; 2) objective cognitive impairment in at least one cognitive domain at neuropsychological testing; 3) a Clinical Dementia Rating score of ≤1 and a Mini‐Mental State Examination (MMSE) score of ≥20; and 4) vascular lesions on brain MRI that suggest a link between the cognitive deficit and cSVD. To obtain a range in structural abnormalities, we included healthy aging controls. Controls were matched on age and sex with patients with cSVD and had no overt cerebrovascular diseases and cognitive impairment (MMSE >24).

### *Image Acquisition*

Participants underwent imaging on a 3T MR system (Achieva TX, Philips Healthcare, Best, the Netherlands) employing a 32‐element head coil suitable for parallel imaging. For anatomical reference, a T_1_‐weighted pulse sequence was applied (repetition time / inversion time / echo time [TR/TI/TE] 8.3/800/3.8 msec; field of view [FOV] 256 × 256 × 160 mm^3^; 1.0 mm cubic voxel). To visualize enlarged PVS, a T_2_‐weighted fluid attenuated inversion recovery (FLAIR) pulse sequence (TR/TI/TE 4800/1650/299 msec; FOV 256 × 256 × 180 mm^3^; 1.0 mm cubic voxel) and a T_2_‐weighted pulse sequence (TR/TE 2000/80 msec; FOV 230 × 143 × 184 mm^3^; 1.7 × 1.7 × 5.5 mm^3^ voxel) were employed. IVIM imaging was performed using a Stejskal–Tanner diffusion‐weighted single‐shot spin‐echo echo‐planar imaging (EPI) pulse sequence (TR/TE = 6800/84 msec; FOV 221 × 269 × 139 mm^3^; 2.4 × 2.4 × 2.4 mm^3^ voxel size; acquisition time 5:13 min; anterior–posterior direction) as described previously.^8^ Prior to this sequence an inversion pulse (TI 2230 msec) was applied to suppress the contamination by CSF. Fifteen diffusion‐sensitive *b*‐values (0, 5, 7, 10, 15, 20, 30, 40, 50, 60, 100, 200, 400, 700, and 1000 s/mm^2^) were employed and two and three signal averages were taken for *b*‐value = 700 and 1000 s/mm^2^, respectively, to increase the signal‐to‐noise‐ratio.

### *Regions of Interest (ROIs)*

The brain was automatically segmented (Freesurfer^9^) from which the white matter, cortex, and deep gray matter could be selected. WMHs were identified on a FLAIR image using a semiautomated algorithm^10^ followed by visual corrections including the identification of lacunar infarcts. Subsequently, the white matter was divided into normal‐appearing white matter (NAWM) and WMHs. The basal ganglia (BG) and centrum semiovale (CSO) were selected as ROIs, where PVS likely are present. BG was obtained by selecting the caudate, putamen, pallidum, accumbens, and substantia nigra and the CSO was defined as the NAWM superior to the lateral ventricles. Lesions were excluded from both the BG and CSO. This resulted in the ROIs: BG, CSO, WMHs, NAWM, and cortex.

### *Rating of Lesions*

Enlarged PVS were rated by three raters (R.J.v.O., J.S., and P.H.M.V., with 20, 10, and 0 years of experience, respectively) as previously described.^11^ Briefly, enlarged PVS were defined as round‐ or oval‐shaped lesions (<3 mm) or linear‐shaped lesions. Lesions had a smooth margin, absence of mass effect, and signal intensity equal to CSF on T_2_‐weighted images. Moreover, when concurrently visible on FLAIR images, lesions would appear hypointense without a hyperintense rim.^12^ One slice and one hemisphere showing the most PVS were selected for rating. Enlarged PVS were scored separately for the BG and CS. Participants were divided into three categories: low (number of PVS ≤10), moderate (10 < number of PVS < 25), and high (number of PVS ≥25). The intraclass correlation coefficient for the three raters was 0.73 (95% confidence interval [CI] 0.61–0.82) and 0.77 (95% CI 0.66–0.84), for BG and CS, respectively.

The total WMHs volume was calculated by taking the sum of all voxels identified as WMHs on FLAIR images and multiplied by the voxel volume (ie, 1 mm^3^). Subsequently, the relative WMH volume was calculated by dividing the WMH volume by the intracranial volume. Subjects with a very low number of WMHs voxels (ie, less than 20 voxels of WMHs) were excluded.

### *IVIM Analysis*

#### *Image Preprocessing*

IVIM images were corrected for head displacements, EPI, and eddy current distortions (ExploreDTI v. 4.8.3). Furthermore, they were aligned with T_1_‐weighted images and spatially smoothed with a 3 mm full‐width‐at‐half‐maximum Gaussian kernel. Cross‐modality registration was performed using FLIRT from the FMRIB's Software Library (FSL) v. 5.0.4 (https://fsl.fmrib.ox.ac.uk/fsl/fslwiki).

#### *IVIM Curve Fitting*

Traditionally, the IVIM signal is assumed to decay biexponentially,^2^ which arises from two diffusivity components: the parenchymal diffusivity *D* and intravascular diffusivity D*. The corresponding signal decay equation, which accounts for the applied inversion pulse and differences in longitudinal and transverse relaxation times in tissue and blood, can be expressed as^13^:

(1) $$\frac{S\left(b\right)}{S\left(0\right)}=\frac{\left(1-f_{\text{perf}}\right)E_{1,\mathrm{par}}E_{2,\mathrm{par}}e^{-\mathrm{bD}}+f_{\text{perf}}\;E_{1,\text{perf}}E_{2,\text{perf}}\;e^{-b\left(D+D^{*}\right)}\;}{\left(1-f_{\text{perf}}\right)E_{1,\mathrm{par}}E_{2,\mathrm{par}}+f_{\text{perf}}\;E_{1,\text{perf}}E_{2,\text{perf}}}$$

where *S(b)* represents the signal intensity at *b*‐value *b*, *f*_perf_ the microvascular perfusion fraction, *E*_*1,k*_ and *E*_*2,k*_ denote the longitudinal and transverse correction, respectively, for a specific compartment *k*. We introduce these terms to facilitate easy notation.

*E*_*1,k*_ for the parenchymal and intermediate diffusion compartment is defined as:

(2) $$E_{1,k}=\left(1-2e^{-\frac{\mathrm{TI}}{T_{1,k}}}+e^{-\frac{\mathrm{TR}}{T_{1,k}}}\right),\text{with}\;k=\mathrm{par}\;\text{or}\;\mathrm{int}$$

In the case of flow, *E*_*1,k*_ for the microvascular perfusion compartment can be defined as:

(3) $$E_{1,\text{perf}}=\left(1-e^{-\frac{\mathrm{TR}}{T_{1,\text{perf}}}}\right).$$

*E*_*2,k*_ for all three compartments is defined as:

(4) $$E_{2,k}=e^{-\frac{\mathrm{TE}}{T_{2,k}}}\;k=\mathrm{par},\mathrm{int}\;\text{or perf}$$

#### *NNLS*

The NNLS method has previously been employed to perform multiexponential signal analysis.^14^ The signal is considered to be comprised of the sum of multiple components decaying exponentially with diffusion coefficient *D*, which can be represented in a discretized form as follows:

(5) $$\frac{S\left(b_{i}\right)}{S\left(0\right)}=\sum_{j=1}^{M}{\dot{a}}_{j}∙e^{-b_{i}D_{j}},i=1,\ldots ..,N$$

where *D*_*j*_ is the *j*‐th diffusion coefficient, ${\dot{a}}_{j}$ the pertaining (relative signal) amplitude of the basis function $e^{-b_{i}D_{j}}$, *M* the number of diffusion coefficients between the imposed *D*_min_ and *D*_max_, and *N* the number of *b*‐values. Note that no assumptions were made concerning the number of components. Subsequently, Eq. 5 can be numerically solved in a least‐squares fashion. Here it is assumed that ${\dot{a}}_{j}$ is nonnegative (ie, ${\dot{a}}_{j}$ ≥0), because a negative ${\dot{a}}_{j}$ has no physiological meaning. Furthermore, a dictionary of *D* values was used ranging from *D*_min_ of 0.1·10^‐3^ and *D*_max_ of 1000·10^‐3^ mm^2^/s with 200 logarithmically scaled values.

#### *Specification of* D *Ranges*

**Figure 2:**
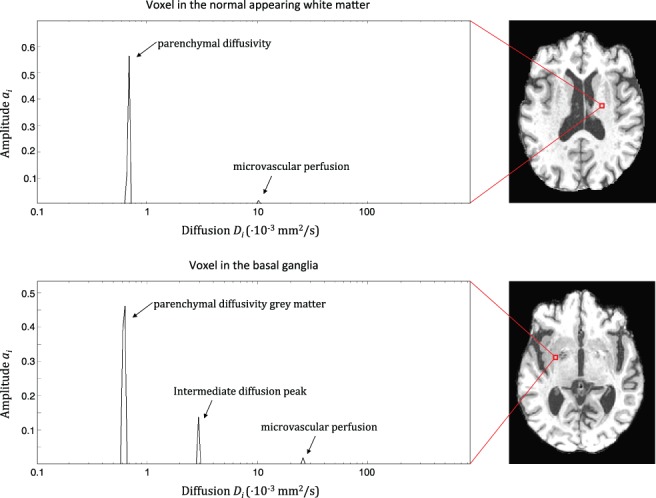
Diffusion spectra in which the diffusion coefficient *D*_*i*_ with corresponding amplitude *a*_*i*_ is depicted for a voxel in the normal‐appearing white matter (upper image) and in basal ganglia (lower image). A parenchymal and microvascular perfusion component can be distinguished in both spectra. In the spectra obtained from a voxel in the basal ganglia an intermediate diffusion component can be observed in between the parenchymal and microvascular components.

Figure 2 shows typical diffusion spectra of voxels in the NAWM and BG in patients with cSVD obtained using NNLS. The spectrum was divided into three ranges based on the observed locations of the peaks. In both diffusion spectra, a peak with 0.1 < *D* < 1.5·10^‐3^ mm^2^/s can be seen which corresponds with water diffusion in parenchyma^14^, ^15^ (ie, parenchymal diffusivity). Furthermore, another peak can be observed in the range 4.0 < *D* < 1000·10^‐3^ mm^2^/s with intravascular diffusivity values at least 10 times higher than parenchymal diffusivity,^2^ which are termed microvascular perfusion. Moreover, in the spectrum obtained within BG, an additional peak in between the parenchymal and microvascular perfusion components (1.5 ≤ *D* ≤ 4.0·10^‐3^ mm^2^/s) can be observed. This peak is denoted as the intermediate diffusion component *D*_int_. We hypothesized that in lesion‐prone regions this peak is indicative of an aberrant amount of ISF, which has similar diffusion properties as CSF (*D* = 3.0·10^‐3^ mm^2^/s), but shorter T_1_ relaxation time. Due to the shorter T_1_ we assume that the ISF is not suppressed by the inversion pulse, unlike CSF.

#### *Quantitative Measures*

To assess the contribution of the intermediate diffusion component to the IVIM signal in a voxel, the fraction of this component was quantified. We assumed that the component represents a (physical) compartment and hence the quantified fraction is representative for the volume fraction of the compartment in a voxel. Hereafter, compartment instead of component will be used when mentioned in the same context as the volume fraction. The fraction of the intermediate diffusion compartment is defined as:

(6) $$f_{\mathrm{int}}=C\;A_{\mathrm{int}}$$

where *f*_int_ is the volume fraction of the intermediate diffusion compartment, *C* is a correction factor accounting for the inversion pulse and different longitudinal and transverse time of the compartment, and *A*_int_ is the sum of (relative signal) amplitudes calculated by integrating over all amplitudes in the intermediate diffusion *D* range divided by the sum of all amplitudes.

#### *Presence of Three Compartments*

To quantify the volume fractions, the sum of amplitudes of the parenchymal *A*_par_, the intermediate diffusion *A*_int_, and microvascular perfusion compartment *A*_perf_ was calculated by integrating over all amplitudes in the corresponding *D* range divided by the total integral over all amplitudes of the complete spectrum. To account for the inversion pulse and effects of longitudinal and transverse relaxation times for a specific compartment, the amplitude *A*_*k*_ for a specific compartment *k* is represented by the following equation:

(7) $$A_{k}=\frac{f_{k}∙E_{1,k}∙E_{2,k}}{\sum_{k=1}^{3}f_{k}∙E_{1,k}∙E_{2,k}}\;$$

where *f*_*k*_ is the volume fraction for a specific compartment *k*, *E*_*1,k*_ and *E*_*2,k*_ the transverse and longitudinal correction (Eqs. 2, 3, 4), respectively, for a specific compartment. In the presence of peaks in all three compartments *f*_par_ *, f*_int_, and *f*_perf_ can be solved by using Eq. 7 to formulate the following expression:

(8) $$\frac{f_{\mathrm{par}}∙E_{1,\mathrm{par}}∙E_{2,\mathrm{par}}}{A_{\mathrm{par}}}=\frac{f_{\mathrm{int}}∙E_{1,\mathrm{int}}∙E_{2,\mathrm{int}}}{A_{\mathrm{int}}}=\frac{f_{\text{perf}}∙E_{1,\text{perf}}∙E_{2,\text{perf}}}{A_{\text{perf}}}.$$

The terms *f*_par_ *, f*_int_, and *f*_perf_ denote the volume fractions, which are present within 1 voxel, and by definition add up to 1. *f*_par_ denotes the fraction that can be attributed to the parenchyma, *f*_int_ is the intermediate volume fraction, and *f*_perf_ is the blood perfusion fraction.

The expression above was obtained by using the fact that a similar denominator is shared in Eq. 7 when calculating the amplitude for each compartment. Assuming that the sum of *f*_par_ *, f*_int_, and *f*_perf_ is equal to 1, this leads to:

(9) $$f_{\mathrm{par}}=\frac{E_{1,\mathrm{int}}E_{1,\text{perf}}E_{2,\mathrm{int}}E_{2,\text{perf}}}{d}\;A_{\mathrm{par}},$$

(10) $$f_{\mathrm{int}}=\frac{E_{1,\mathrm{par}}E_{1,\text{perf}}E_{2,\mathrm{par}}E_{2,\text{perf}}}{d}\;A_{\mathrm{int},}$$

and

(11) $$f_{p}=\frac{E_{1,\mathrm{par}}E_{1,\mathrm{int}}E_{2,\mathrm{par}}E_{2,\mathrm{int}}}{d}\;A_{\text{perf}}$$

with

(12) $$d=A_{\mathrm{par}}E_{1,\mathrm{int}}E_{1,\text{perf}}E_{2,\mathrm{int}}E_{2,\text{perf}}+A_{\mathrm{int}}E_{1,\mathrm{par}}E_{1,\text{perf}}E_{2,\mathrm{par}}E_{2,\text{perf}}\;+A_{p}E_{1,\mathrm{par}}E_{1,\mathrm{int}}E_{2,\mathrm{par}}E_{2,\mathrm{int}}$$

#### *Presence of Two Compartments*

To provide a better, educational insight, let us assume that only two compartments *k* = 1 and *k* = 2 (eg, 1 = par and 2 = int, 1 = par and 2 = perf or 1 = int and 2 = perf) are present in the diffusion spectrum. The volume fractions *f*_1_ and *f*_2_ are quantified using the following expression:

(13) $$\frac{f_{1}∙E_{1,1}∙E_{2,1}}{A_{1}}=\frac{f_{k2}∙E_{1,2}∙E_{2,2}}{A_{2}}$$

Assuming that the sum of two compartments equals 1, leads to:

(14) $$f_{1}=\frac{E_{1,2}E_{2,2}}{d}∙A_{1}\;$$

and

(15) $$f_{2}=\frac{E_{1,1}E_{2,1}}{d}∙A_{2}\;$$

with

(16) $$d=A_{1}E_{1,2}E_{2,2}+A_{2}E_{1,1}E_{2,2}\;$$

For the longitudinal and transverse relaxation time of parenchyma and blood, literature values were used: T_1_,par = 1081 msec, T_2_,par = 95 msec, T_1,perf_ = 1624 msec and T_2,perf_ = 275 msec.^15^, ^16^, ^17^

### *T_1_ of the Intermediate Diffusion Compartment*

To estimate the T_1_ of the intermediate diffusion compartment an iterative calculation process was performed. The following relation was used in the case of three compartments:

(17) $$\frac{1}{T_{1,\mathrm{vox}}}=f_{\mathrm{par}}∙\frac{1}{T_{1,\mathrm{par}}}+f_{\mathrm{int}}∙\frac{1}{T_{1,\mathrm{int}}}+f_{\text{perf}}∙\frac{1}{T_{1,\text{perf}}}$$

where T_1,vox_ is the T_1_ of the entire voxel calculated using T_1_‐mapping which has been described previously.^6^, ^18^ The iterative processes is initialized with calculating start values of *f*_par_ *, f*_int_, and *f*_perf_ by using a value for T_1,0,int_ of 3000 msec and T_2,0,int_ of 1500 msec in Eq. 9, 10, 11, 12. Here, the initial values of T_1_ and T_2_ of the intermediate diffusion component were chosen close to free water, since we assume that the intermediate diffusion component may have comparable properties to free water, as indicated previously. Subsequently, the resulting *f*_par_ *, f*_int_, and *f*_perf_ values are used in Eq. 17 to calculate T_1,int._ Hereafter, T_1,int_ was used to recalculate *f*_par_ *, f*_int_, and *f*_perf_ in Eqs. 9, 10, 11, 12. This process is reiterated until the change in T_1,int_ between iterations is smaller than 10 msec. In case no convergence of the change in T_1,int_ takes place, an average T_1,int_ from nearby voxels that did converge, was used. A similar process is carried out when two compartments (ie, intermediate diffusion and microvascular compartment or intermediate diffusion and parenchymal compartment) are present. Note that calculation of a T_1,int_ is only possible when the intermediate diffusion compartment is observed in the spectrum.

Furthermore, *D*_int_ was calculated for all ROIs by taking the geometrical mean *D* in the corresponding *D* range. Both *f*_int_ and *D*_int_ were calculated in a voxelwise manner and averaged per ROI. In addition to its relative volume fraction, the IVIM technique allows estimating ISF dynamics, ie, the diffusion coefficient and T_1_ relaxation time, which both will decrease as function of amount of (waste) solutes.

### *Statistical Analysis*

To assess the presence of the intermediate diffusion component, the occurrence of various types of spectra were tallied and explored. The relations between *f*_int_ with enlarged PVS score in BG and CSO, and with WMH volume in the WMHs, were studied using the Spearman's rank order correlation *r*_s_. Furthermore, to compare *f*_int_ values between ROIs, normalized histograms of *f*_int_ for each ROI were calculated and averaged over all participants. To explore the presence of the intermediate diffusion compartment in the NAWM and cortex, the relation between *f*_int_ and age was investigated. All analyses were performed over the entire study population. Significance was inferred when *P* < 0.05 (SPSS v. 22, IBM, Armonk, NY).

## Results

### *BG and CSO*

In Table 1, *f*_int_ and *D*_int_ are presented for all ROIs. In the BG, *f*_int_ was 4.2 ± 1.7% (mean ± SD), 7.0 ± 4.1% and 13.6 ± 7.7% for the groups with a low (*n* = 66 subjects), moderate (*n* = 28 subjects), and high number (*n* = 12 subjects) of enlarged PVS, respectively. In the CSO, *f*_int_ was 3.9 ± 1.3%, 4.4 ± 1.4%, and 4.5 ± 1.2% for the groups with a low (*n* = 36 subjects), moderate (*n* = 46 subjects), and high (*n* = 24 subjects) number of enlarged PVS, respectively. In the BG and CSO, 22.0% and 26.0% of all voxels, respectively, contain a spectrum with an intermediate diffusion component (details of various spectra are given in Tables 2 and 3).

**Table 1:** Intermediate Diffusion Volume Fraction *f*_int_ and Intermediate Diffusivity *D*_intI_

	*f*_int_ (SD) %	*D*_int_ (SD) ·10^‐3^ mm^2^/s
BG	6.0 (4.6)	2.4 (0.2)
CS	4.3 (1.3)	2.5 (0.1)
WMHs	27.1 (13.1)	2.0 (0.2)
NAWM	5.0 (1.4)	2.4 (0.1)
Cortex	4.3 (1.2)	2.5 (0.1)

BG = basal ganglia; CS = centrum semiovale; WMHs = white matter hyperintensities; NAWM = normal appearing white matter.

**Table 2:** Occurrence of Spectra in % (SD) With Various Combinations of Components

Tissue region	Par only	Perf only	Par & Perf	Spectra with Int
BG	21.1 (11.6)	2.0 (2.0)	53.4 (13.3)	22.0 (8.0)
CS	6.2 (5.6)	0.4 (0.7)	67.3 (8.4)	26.0 (6.6)
WMHs	7.5 (11.8)	10.9 (7.1)	36.2 (19.8)	42.2 (18.3)
NAWM	12.8 (8.0)	1.8 (0.8)	62.2 (6.8)	22.5 (4.1)
Cortex	11.9 (11.6)	0.9 (2.0)	66.0 (6.5)	20.9 (4.3)

Par = parenchymal component; Int = Intermediate diffusion component; Perf = perfusion component; BG = basal ganglia; CS = centrum semiovale; WMHs = white matter hyperintensities; NAWM = normal‐appearing white matter.

**Table 3:** Occurrence of Spectra in % (SD) in Which an Intermediate Diffusion Component Is Present

Tissue region	Par, Int & Perf	Par & Int	Int & Perf	Int only
BG	45.2 (14.5)	46.1 (13.7)	5.0 (6.8)	3.7 (5.8)
CS	74.2 (14.2)	25.5 (14.1)	0.2 (0.9)	0.1 (0.3)
WMHs	43.3 (19.5)	26.2 (17.2)	21.7 (13.9)	8.9 (8.7)
NAWM	62.5 (11.4)	32.4 (10.9)	3.3 (2.0)	1.7 (1.6)
Cortex	65.6 (11.5)	29.7 (10.6)	3.6 (2.4)	1.1 (1.1)

Par = parenchymal component; Int = Intermediate diffusion component; Perf = perfusion component; BG = basal ganglia; CS = centrum semiovale; WMHs = white matter hyperintensities; NAWM = normal‐appearing white matter.

Note that in this table only spectra containing an intermediate diffusion component are considered.

**Figure 3:**
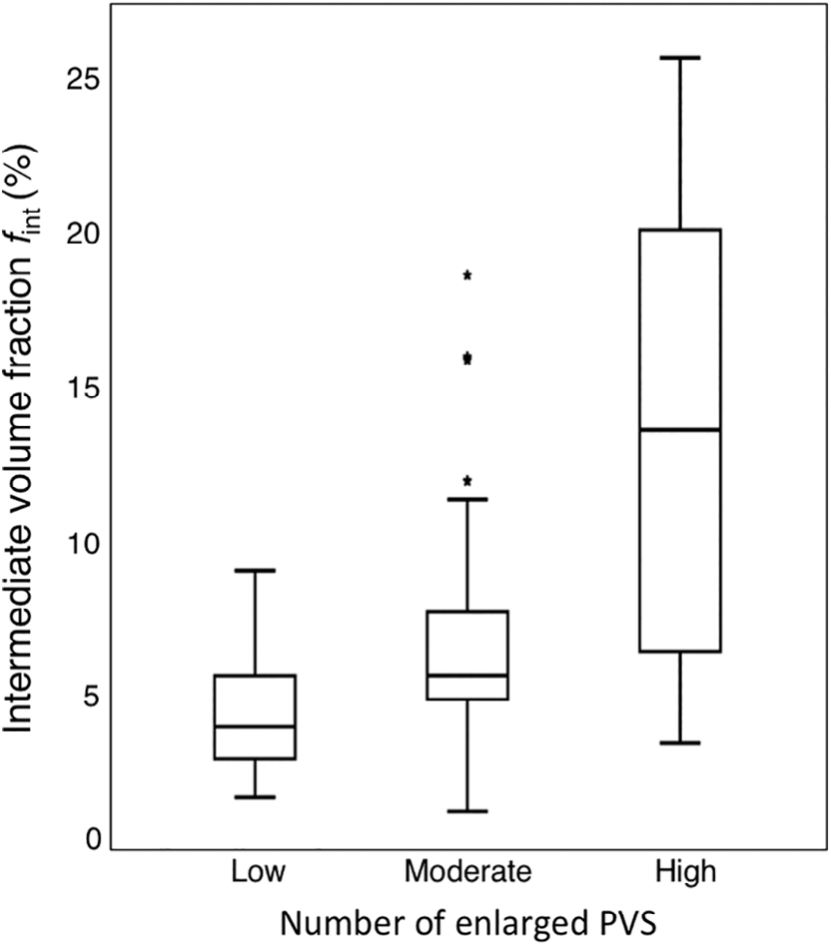
Boxplots of the intermediate diffusion volume fraction for the groups with low, moderate, and high number of enlarged PVS in the basal ganglia. A larger *f*_int_ was significantly correlated (*r*_s_ = 0.49, *P* < 0.01) with a higher number of enlarged PVS.

**Figure 4:**
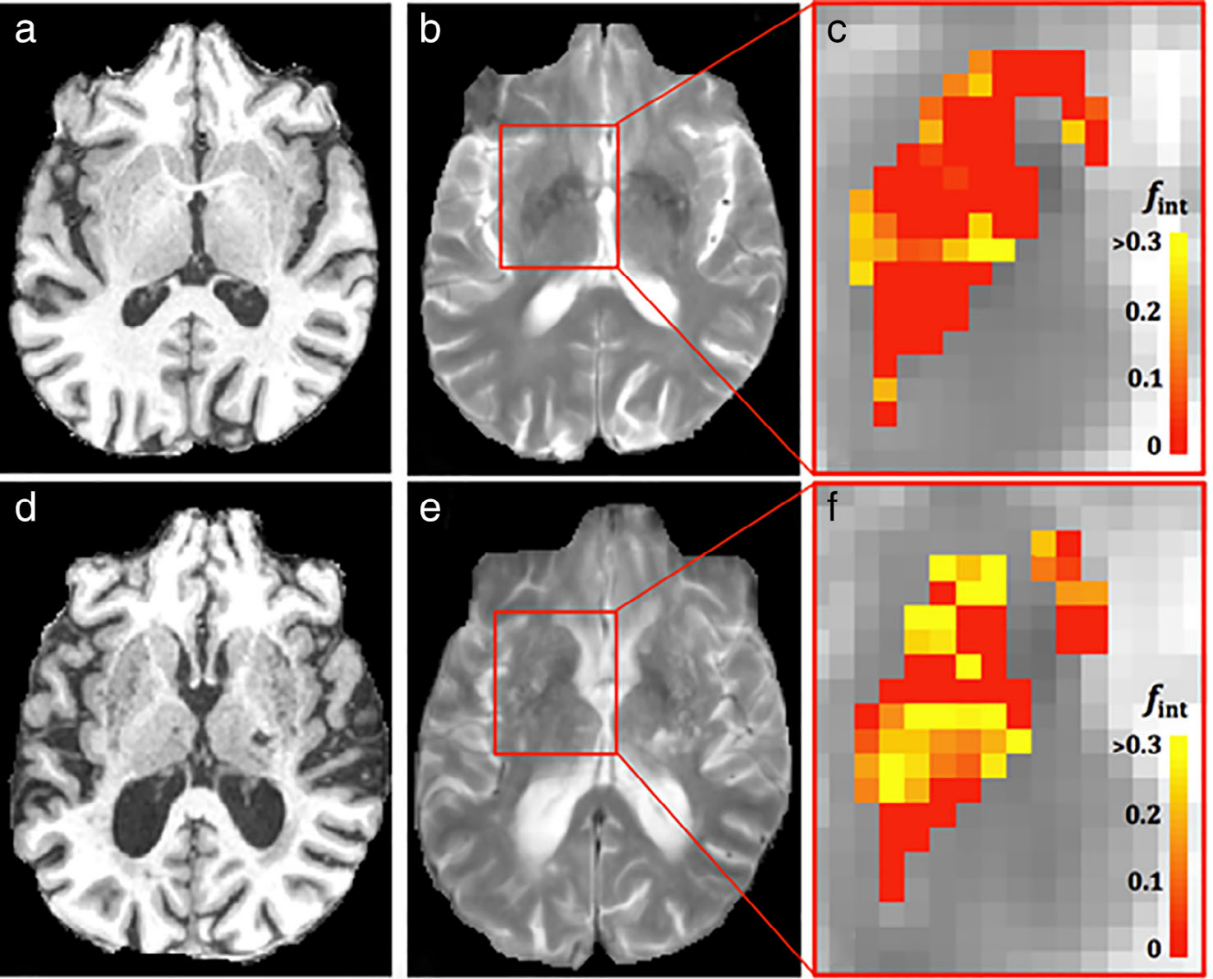
T_1_‐weighted, T_2_‐weighted images, and maps of intermediate diffusion volume fraction in the basal ganglia for a participant (male, 67 years) with low (**a‐c**, respectively) and a participant (female, 84 years) with high number of enlarged PVS (**d–f**, respectively). More voxels with higher values of *f*_int_ can be observed for the participant with a higher number of visible PVS.

A larger *f*_int_ in both the BG (*r*_s_ = 0.49, *P* < 0.01) and CSO (*r*_s_ = 0.23, *P* = 0.02) was significantly correlated with a higher number of enlarged PVS (Fig. 3 for BG). Maps of *f*_int_ are shown in Fig. 4.

The average T_1_ for the intermediate diffusion component was 1.25 ± 0.27 sec.

### *WMHs*

**Figure 5:**
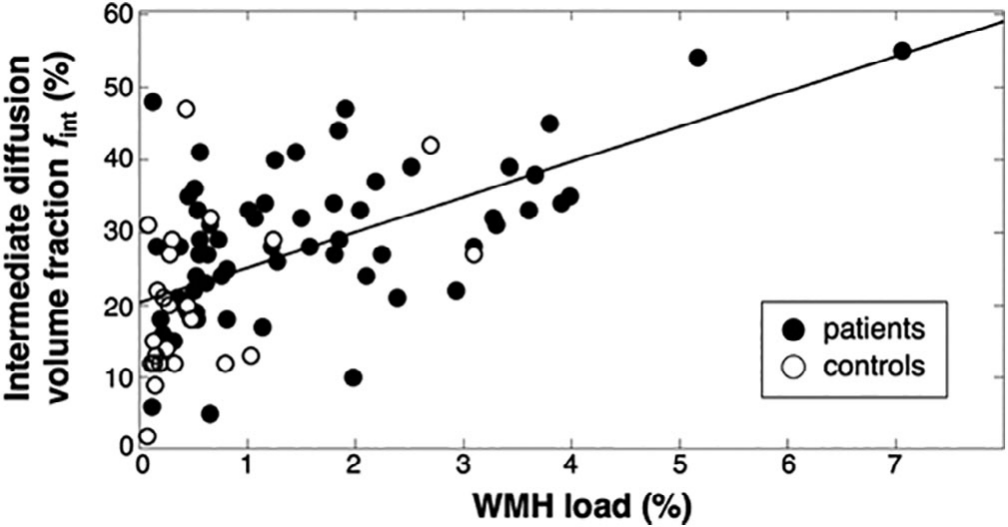
Relation between the intermediate diffusion volume fraction in the WMHs and relative WMH volume. A significant correlation was observed (*r*_underscore(s) = 0.57, *P* < 0.01). Please note that the regression line is only added for visualization.

In the WMHs, the largest percentage of brain tissue voxels containing spectra with an intermediate diffusion component (42.2%) and largest *f*_int_ (27.14 ± 13.13%) can be found compared with other ROIs (Tables 1 and 2). The WMH volume for 64 patients with cSVD was 1.5 ± 1.4% and for 24 controls was 0.6 ± 0.8% relative to the total intracranial volume. In Fig. 5, the WMH volume was plotted against *f*_int_. A significant increase of *f*_int_ with increasing WMH volume was found (r_s_ = 0.57, *P* < 0.01).

### *Normal‐Appearing Tissue and Relation With Age*

**Figure 6:**
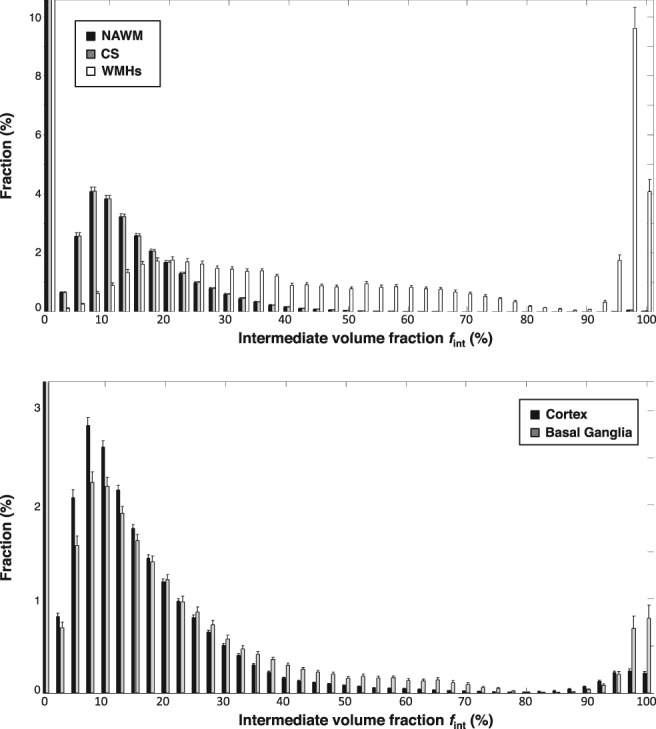
Normalized histograms of the white matter (top) divided into the normal‐appearing white matter (NAWM) (black), centrum semiovale (CS) (gray), and white matter hyperintensities (WMHs) (white), and gray matter (bottom) divided into the cortex (black), and basal ganglia (gray). Higher values *f*_int_ in the WMHs can be observed than in NAWM and CS, which originates from spectra without a parenchymal compartment. Additionally, higher values of *f*_int_ can be observed in the basal ganglia than in the cortex. Note that we magnified certain regions of the histograms to better visualize bins with lower fractions and hence fractions larger than 10.4% and 3.2% are not shown, for white matter and gray matter, respectively.

**Figure 7:**
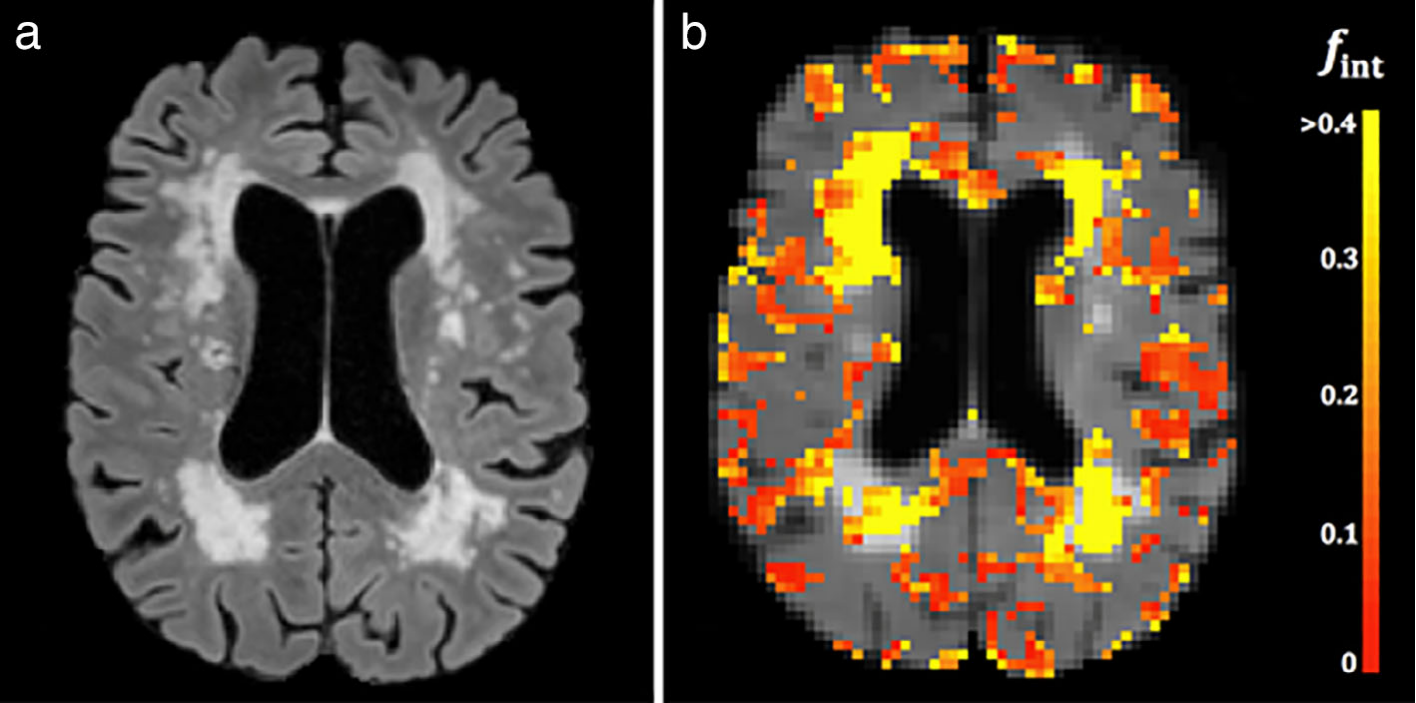
FLAIR image **(a)** and superimposed color‐coded map of intermediate diffusion volume fractions **(b)** of a patient (male, 79 years) with cerebral small vessel disease. High values of intermediate diffusion volume fractions strongly colocalize with white matter hyperintensities. Please note that pericortal regions with elevated intermediate diffusion volume fractions are likely due to partial volume effects. For display purposes, the CSF in the ventricle has been masked out.

Also, the NAWM and cortex contain tissue voxels that show spectra with an intermediate diffusion component (range: 20.9–22.5%, Table 2). We obtained a significant correlation between *f*_int_ in both the NAWM (*r*_p_ = 0.37, *P* < 0.01) and cortex (*r*_*p*_ = 0.24, *P* = 0.01) with age. In Fig. 6, normalized histograms are shown and in Table 1 mean values of *f*_int_ are given. Intermediate diffusion volume fractions *f*_int_ were significantly lower for the NAWM than WMHs (*P* < 0.01) and CSO (*P* < 0.01). Furthermore, *f*_int_ for the cortex was lower than for the BG (*P* < 0.01). Figure 7 shows a map of *f*_int_ for one slice of the entire brain. It can be appreciated that voxels with high *f*_int_ values strongly colocalized with WMHs.

## Discussion

In the current study we employed IVIM imaging and spectral analysis using the NNLS approach to study the presence of an additional diffusion component. First, we detected an intermediate diffusion component, which lies in between the traditionally assumed parenchymal diffusion and microvascular components. This component was found in healthy aging tissue as well as in tissue regions prone to the occurrence of WMHs and MRI‐visible enlarged PVS. Second, we showed that the intermediate diffusion volume fraction *f*_int_ was significantly correlated with both the number of enlarged PVS and WMH volume. Third, the intermediate diffusion component in normal‐appearing tissue (ie, NAWM and cortex) had lower volume fractions than in regions where structural abnormalities of cSVD are expressed (ie, BG, CSO, and WMHs).

In our study, we demonstrated that the intermediate diffusion volume fraction increased with the extent of enlarged PVS and WMH volume. This relation can be explained as follows. In enlarged PVS, relatively large amounts of ISF are present, which has been suggested to indicate perivascular edema caused by an accumulation of blood‐borne products through an impaired blood–brain barrier (BBB), one of the pathophysiological mechanisms involved in cSVD.^3^, ^19^, ^20^, ^21^ Perivascular edema may be toxic for brain cells and can ultimately result in neuronal damage leading to demyelination, which is typical for WMHs.^20^, ^22^, ^23^ Hence, perivascular edema may precede tissue degeneration. In addition, an unusual amount of ISF is also observed in the WMHs, which is likely related to the loosening of the white matter tracts, the reduction in the number of oligodendrocytes, subtotal loss of myelin and axons,^24^, ^25^ and degeneration of the extracellular matrix.^26^ Due to the increased space with ISF in impaired tissue, water diffusion is less hindered than in healthy tissue, and a distinct component with intermediate diffusion properties becomes apparent. Therefore, we argue that the presence of the intermediate diffusion component is indicative of the increased amount of ISF, which can diffuse more freely in regions with enlarged PVS and WMHs. Additionally, the T_1_ of the intermediate diffusion volume fraction was in the expected range (ie, between T_1_ of parenchyma and CSF), and therefore likely indicative of an increased amount of (waste) solutes.

This could be indicative of an impaired glymphatic system, which is a waste clearance mechanism named after its involvement of glial cells and its functional resemblance to the lymphatic system.^27^ The glymphatic system utilizes a system of connecting PVS, to efficiently eliminate soluble proteins and metabolites from the central nervous system. It has been suggested that glymphatic dysfunction represents a fundamental constituent of cSVD.^3^ Although it has previously been shown in the rat brain that diffusion MRI has the potential for the assessment of perivascular fluid motion in relation to the glymphatic system,^28^ whether the current clinical application of NNLS‐based IVIM can accurately detect glymphatic failure remains to be investigated. For this, future studies should combine these measures with assessments of cardiac pulsatility (eg, pulsatility of small perforating lenticulostriatal arteries^29^) and aquaporin‐4 dependent fluid movement.^30^ Additionally, it would be interesting to compare the NNLS approach with methods that can separate diffusion properties of brain tissue from surrounding free water while mapping the free water volume.^31^

The intermediate diffusion volume fractions could also be detected in normal‐appearing tissue (ie, NAWM and cortex), although these fractions were lower than those of regions with structural abnormalities (ie, BG, CSO, and particularly WMHs). We found a significant correlation between this volume fraction in normal‐appearing tissue and age, which indicates an effect of normal aging. This is in line with the association between decreasing tissue integrity,^32^ PVS enlargement,^33^ and the declining clearance process of ISF by the glymphatic system^34^ with aging.

The association of the intermediate diffusion component *f*_int_ with enlarged PVS was stronger for the BG than for CSO, which could be due to a denser vasculature of the BG.^35^ Furthermore, it has previously been suggested that different pathophysiological processes might underlie enlarged PVS in the BG and CSO.^36^

Previously, other models have been proposed to extend the two‐compartment approach, including three‐compartment models to separate the parenchymal, microvascular perfusion, and an additional component which is suggested to indicate freely diffusing water,^37^, ^38^ a model which decomposes the microvascular flow into a fast and slow component^39^ and a model that disentangles diffusive and ballistic parts.^40^ These models differ from our approach. We argue that when fewer components are present than assumed by these models, overfitting can potentially confound results. In contrast, the NNLS method does not infer the number of components and is less sensitive to overfitting. Nevertheless, a dedicated comparison between the NNLS method and models with a fixed number of components is needed to compare their performances, preferably in animal models where tissue specimens can be validated microscopically.

Our study encompasses several strengths. First, the applied IVIM method is widely available on clinical MRI scanners, making the transition into the clinic feasible. Second, fitting the curve with the NNLS method does not make any assumptions on the number of components. Third, we performed this method directly in a patient population for whom it can be relevant; the intermediate diffusion volume fraction can potentially aid in providing an early disease marker for cSVD. Fourth, we employed an inversion pulse to suppress the contamination of CSF, which harbors a fully freely diffusing component. Lastly, our model accounts for the influence of relaxation time for various components to estimate the actual volume fraction of the intermediate compartment rather than the signal fraction.

This study has also some limitations. The assumed one‐to‐one relation between exponential components and microscopic compartments and the definition of the compartments can influence the absolute value of the volume fractions. Also, physiological processes such as cardiac pulsatility might influence the magnitude of the volume fractions.

Our approach assumes T_1_ and T_2_ relaxation properties from the literature, and that these are regionally invariant within specific compartments. Future studies should obtain accurate, quantitative relaxation estimates that also incorporate regional differences.

Furthermore, IVIM imaging was only performed in one direction. Recently, it has been demonstrated that the vascular components are dependent on the diffusion‐sensitizing direction, which might have an effect on the absolute quantification of the intermediate diffusion volume fraction.^41^ Nevertheless, our study performed in one diffusion‐sensitizing direction offers preliminary results showing the merit of the NNLS method in analyzing the IVIM signal in cSVD.

In conclusion, using the NNLS method in IVIM we identified and quantified an intermediate diffusion component in cSVD. We showed the relation between the intermediate volume fraction with WMHs and enlarged PVS and argued that it could potentially be indicative of aberrant amounts of ISF in degenerated tissue or perivascular edema preceding tissue degeneration. Moreover, we demonstrated that the intermediate diffusion volume fraction was also present (although with lower values) in normal‐appearing tissue of patients with cSVD and the healthy elderly. Hence, the intermediate diffusion volume fraction can potentially function as a quantitative imaging biomarker of tissue degeneration in cSVD, potentially linked to glymphatic dysfunction. Longitudinal studies are required to further investigate the precise nature of the intermediate diffusion volume fraction.
